# Nomograms based on the lymphocyte–albumin–neutrophil ratio (LANR) for predicting the prognosis of nasopharyngeal carcinoma patients after definitive radiotherapy

**DOI:** 10.1038/s41598-024-56043-z

**Published:** 2024-03-05

**Authors:** Sujuan Zhang, Zui Chen, Jie Ling, Yuhua Feng, Yangchun Xie, Xianling Liu, Chunhong Hu, Tao Hou

**Affiliations:** Department of Oncology, The Second Xiangya Hospital, Central South University, Changsha, 410011 Hunan China

**Keywords:** Lymphocyte, Neutrophil, Albumin, Nasopharyngeal carcinoma, Prognosis, Head and neck cancer, Tumour biomarkers, Oncology, Cancer

## Abstract

Much evidence has accumulated to show that inflammation and nutritional status are associated with the prognosis of patients with various cancers. The present study was designed to explore the prognostic role of the LANR in NPC patients receiving definitive radiotherapy and to construct a nomogram for predicting patient survival. This study retrospectively reviewed 805 NPC patients (604 in the training cohort and 201 in the validation cohort) who received definitive radiotherapy between January 2013 and December 2019. The clinical data and pretreatment laboratory test data, including lymphocyte count, neutrophil count, and serum ALB concentration, were collected for all patients. The LANR was calculated as the albumin × lymphocyte/neutrophil ratio. Patients in the training cohort and validation cohort were categorized into high-LANR and low-LANR groups according to the corresponding cutoff values. The independent prognostic factors for overall survival (OS), progression-free survival (PFS), relapse-free survival (RFS), and metastasis-free survival (MFS) were evaluated by univariate and multivariate Cox regression analyses, and a nomogram was subsequently constructed. The performance of the nomogram was evaluated by the concordance index (C-index) and calibration curve. A low LANR (< 14.3) was independently associated with worse OS, PFS and MFS in NPC patients. A prognostic prediction nomogram was established based on T stage, N stage, Eastern Cooperative Oncology Group (ECOG) score, treatment modality, and LANR and was validated. The C-indices of the nomograms for OS and PFS in the training cohort were 0.729 and 0.72, respectively. The C-indices of the nomograms for OS and PFS in the validation cohort were 0.694 and 0.695, respectively. The calibration curve revealed good consistency between the actual survival and the nomogram prediction. Patients with NPC with low pretreatment LANR had a poor prognosis. The nomogram established on the basis of the LANR was efficient and clinically useful for predicting survival in NPC patients who underwent definitive radiotherapy.

## Introduction

Nasopharyngeal carcinoma (NPC) is a common head and neck cancer in southern China and southeast Asia^1^. Radiotherapy is the pillar of treatment. With the development of radiotherapy and medical treatments, the prognosis of NPC patients, especially locally advanced NPC patients, has significantly improved^2^. However, approximately 10% of patients will suffer from relapse, and 20% will suffer from metastasis^3^. The TNM staging system is the most prevalent prognostic system used in clinical practice. However, the TNM staging system is not sufficient for accurate differentiation due to the heterogeneity of patients. Thus, the exploration of additional biomarkers for make personalized and accurate predictions is challenging and serves an unmet clinical need.

Promoting inflammation and deregulating metabolism are widely accepted hallmarks of cancer^4^. Numerous studies have demonstrated a significant correlation between inflammatory and nutritional biomarkers and patient prognosis in various types of tumors. Blood immune cells, including neutrophils, lymphocytes, monocytes and platelets, are major mediators of the host inflammatory response and could be used as biomarkers of host immune-inflammation status. Several previous studies have demonstrated that a series of indices, such as the neutrophil-to-lymphocyte ratio (NLR), platelet-to-lymphocyte ratio (PLR), and systemic immune-inflammation index (SII), are based on blood cell counts and can predict patient prognosis in various cancers, including NPC^5–7^. The serum ALB concentration is the classical biomarker for nutritional status, and previous studies have shown that it is a prognostic factor in various malignancies^8–10^. The LANR is a novel biomarker composed of the serum ALB concentration and the NLR. The LANR is an independent prognostic indicator for patients with resectable colorectal cancer^11^. However, the prognostic role of the LANR in NPC patients has not yet been reported.

In the present study, we retrospectively analyzed the prognostic value of the LANR and other nutritional and inflammation indices in a cohort of NPC patients. A nomogram was constructed to establish a prediction model for the prognosis of NPC patients.

## Methods and materials

### Patients

The data from patients diagnosed with NPC who underwent definitive radiotherapy with or without chemotherapy at the Second Xiangya Hospital, Central South University, from January 2013 to December 2019 were retrospectively analyzed. The inclusion criteria were as follows: (1) had histologically diagnosed NPC, (2) had complete clinicopathological data and laboratory test results, and (3) lacked distant metastasis. The exclusion criteria were as follows: (1) incomplete follow-up data; (2) received anticancer therapy before admission; (3) had a history of chronic inflammatory diseases or other malignancies; and (4) had a recent acute infectious disease. All patients recruited were randomly allocated to a training cohort (n = 604) and a validation cohort (n = 201) at a ratio of 3:1. This research was carried out in accordance with the Declaration of Helsinki and approved by the Ethics Committee of the Second Xiangya Hospital of Central South University. The requirement for written informed consent was waived by the ethical committee of the Second Xiangya Hospital, Central South University.

### Data collection

The demographic and clinical pathological data and laboratory results were obtained from the hospital medical records system. Data regarding patient age, sex, Eastern Cooperative Oncology Group performance status (ECOG PS) score, T stage, N stage, clinical stage, treatment modality, height, body weight, neutrophils, lymphocytes, and serum ALB were collected. The primary biomarker was calculated as follows: LANR = albumin × lymphocyte/neutrophil. All patients were staged according to the 8th edition of the American Joint Committee on Cancer (AJCC) TNM staging system based on enhanced MRI.

### Treatment

The treatment of NPC patients was based on international guidelines. All patients were treated with intensity-modulated radiation therapy (IMRT). The gross tumor volume and clinical tumor volume were defined according to previous guidelines^12^. The prescribed dose was 70 Gy for PGTV, 60 Gy for PTV1 and 54 Gy for PTV2. Radiotherapy was delivered at a dose of 1.82–2.12 Gy daily for 5 days per week. Concurrent chemotherapy (cisplatin or nedaplatin) and targeted therapy (nimotuzumab) were administered according to the stage and tolerability of the patients. Induction chemotherapy (IC) and/or adjuvant chemotherapy (AC) were also given to a proportion of patients according to guideline recommendations and tolerability.

### Follow-up

Patients were followed up by telephone review or inpatient and outpatient medical records. The patients were followed up every 3 months in the first year after the end of treatment, every 3 to 6 months in the second to fifth years after the completion of treatment, and annually after 5 years after the completion of treatment. The primary site was evaluated by enhanced MRI, and distant metastasis was evaluated by enhanced CT and SPECT. PET–CT was used in a proportion of patients when enhanced CT and MRI were not sufficient to make a diagnosis. The last follow-up date was April 30, 2023. Overall survival (OS) was calculated from the date of diagnosis to the date of death from any cause or to the last follow-up date. PFS was defined as the time between the date of diagnosis and the date of disease progression or death. Metastasis-free survival (MFS) was defined as the time between the date of diagnosis and the date of distant metastasis. Relapse-free survival (RFS) was defined as the time between the date of diagnosis and the date of locoregional relapse. The major endpoint of the study was OS.

### Statistical analysis

SPSS statistical software (version 22.0; SPSS, Inc., Chicago, IL, USA) and R software version 4.1.3 (The R Foundation, Vienna, Austria) were used for the data analysis. Receiver operating characteristic (ROC) curves were used to calculate the cutoff value for the LANR. The Chi-square test was used to analyze the associations between LANR and clinicopathological features. The Kaplan–Meier method was used to calculate survival curves. Multivariate analysis was based on the Cox regression model. A two-sided P value < 0.05 was considered indicative of statistical significance. A nomogram was used to visualize the prognostic strength of the indices for OS and PFS. The C-index was defined as the difference between the predicted values of the Cox model in survival analysis and the true values and was used to evaluate the predictive ability of the model, and calibration curves were generated to evaluate the degree of calibration of the clinical prediction models.

## Results

### Patient characteristics

A total of 805 NPC patients were enrolled in this retrospective study; 72.1% were male, and 27.9% were female. The median age was 49 years (range 14–83 years). In the whole cohort, 260 (32.3%) patients received concurrent chemoradiation therapy (CCRT), 545 (67.7%) patients received non-CCRT, 109 patients received radiation therapy concurrent with nimotuzumab, and 436 patients received radiation therapy alone. The median OS was 61 months (IQR 44.4–80.5), and the median PFS was 57.7 months (IQR 40.1–78.9). The 3- and 5-year OS rates of the whole cohort were 87.2% (95% CI 85.4–88.8%) and 78.9% (95% CI 76.7–80.4%), respectively. Among all the patients, 83 (10.3%) had local regional recurrence (primary site recurrence and/or cervical lymph node metastasis), 143 (17.8%) had experienced metastasis, and 195 (24.2%) died. The baseline data of the training cohort (n = 604) and validation cohort (n = 201) are presented in Table 1. The distributions of these baseline characteristics did not significantly differ between the two cohorts (all P > 0.05).

**Table 1:** Clinicopathological characteristics of 805 patients.

Characteristics	Total	Training cohort	Validation cohort	*P* value
	N = 805	N = 604	N = 201	
Age (median [IQR])	48.24 ± 10.71	48.24 ± 10.74	48.22 ± 10.54	0.962
Gender (%)				0.659
Female	224 (27.8%)	171 (28.3%)	53 (26.4%)	
Male	436 (72.2%)	433 (71.7%)	148 (73.6%)	
T stage				0.200
1–2	414 (51.4%)	319 (52.8%)	95 (47.3%)	
3–4	391 (48.6%)	285 (47.2%)	106 (52.7%)	
N stage				0.677
0–1	195 (24.2%)	149 (24.7%)	46 (22.9%)	
2–3	610 (75.8%)	455 (75.3%)	155 (77.1%)	
TNM stage				0.913
I–II	112 (13.9%)	85 (14.1%)	27 (13.4%)	
III–IV	693 (86.1%)	519 (85.9%)	174 (86.6%)	
ECOG performance scale				0.362
0	477 (59.3%)	352 (58.3%)	125 (62.2%)	
1–2	328 (40.7%)	252 (41.7%)	76 (37.8%)	
Treatment				0.602
Non-CCRT	545 (67.7%)	412 (68.2%)	133 (66.2%)	
CCRT	260 (32.3%)	192 (31.8%)	68 (33.8%)	
BMI	23.35 ± 3.12	23.42 ± 3.12	23.14 ± 3.13	0.280
LANR	19.43 ± 8.71	19.41 ± 8.83	19.51 ± 8.37	0.686

*ECOG* Eastern Cooperative Oncology Group, *CCRT* concurrent chemo-radiation therapy, *BMI* body mass index.

### Identification of independent prognostic factors

By setting overall survival status as the endpoint, we used ROC analysis to determine the cutoff value of the LANR. The optimal cutoff value of the LANR was 14.3, with a sensitivity of 73.7% and specificity of 65.1% (Fig. S1). The associations between LANR and the clinicopathological characteristics of the patients are shown in Table 2. LANR status was not associated with any clinicopathological characteristic in the training cohort (all P > 0.05). A low LANR was associated with a higher T stage in the validation cohort (P = 0.028).

**Table 2:** Associations between the LANR and other clinicopathologic characteristics in the training and validation cohorts.

Characteristics	Training set					Validation set				
	LANR		Total	Chi-square	P	LANR		Total	Chi-square	P
	LOW	High				LOW	High			
Age				0.358	0.6145				1.142	0.3602
≥ 60	29 (33.7%)	57 (66.3%)	86			45 (26.0%)	128 (74.0%)	173		
< 60	158 (30.5%)	360 (69.5%)	518			10 (35.7%)	18 (64.3%)	28		
Gender				0.330	0.6255				2.614	0.1501
Male	137 (31.6%)	296 (68.4%)	433			45 (30.4%)	103 (69.3%)	148		
Female	50 (29.2%)	121 (70.8%)	171			10 (18.9%)	43 (81.1%)	53		
T stage				2.387	0.1342				4.914	0.0279
1–2	90 (28.2%)	229 (71.8%)	319			19 (20.0%)	76 (80.0%)	95		
3–4	97 (34.0%)	188 (66.0%)	285			36 (34.0%)	70 (66.0%)	106		
N stage				0.053	0.839				0.283	0.5784
0–1	45 (30.2%)	104 (69.8%)	149			14 (30.4%)	32 (69.6%)	46		
2–3	142 (31.2%)	313 (68.8%)	455			41 (26.5%)	114 (73.5%)	155		
TNM stage				1.193	0.3123				1.228	0.3555
I–II	22 (25.9%)	63 (74.1%)	85			5 (18.5%)	22 (81.5%)	27		
III–IV	165 (31.8%)	354 (68.2%)	519			50 (28.7%)	124 (71.3%)	174		
ECOG performance scale				2.029	0.1806				1.881	0.1933
0	101 (28.7%)	251 (71.3%)	352			30 (24.0%)	95 (76.0%)	125		
1–2	86 (34.1%)	166 (65.9%)	252			25 (32.9%)	51 (67.1%)	76		
Treatment				1.979	0.1858				0.041	0.8689
Non-CCRT	135 (32.8%)	277 (67.2%)	412			37 (27.8%)	96 (72.2%)	133		
CCRT	52 (27.1%)	140 (72.9%)	192			18 (26.5%)	50 (73.5%)	68		
BMI				1.061	0.5883				0.551	0.7592
Underweight	8 (24.2%)	25 (75.8%)	33			3 (33.3%)	6 (66.7%)	9		
Normal weight	100 (32.4%)	209 (57.6%)	309			29 (25.4%)	85 (74.6%)	114		
Overweight	79 (30.2%)	183 (69.8%)	262			23 (29.5%)	55 (70.5%)	78		

*ECOG* Eastern Cooperative Oncology Group, *CCRT* concurrent chemo-radiation therapy, *BMI* body mass index.

**Figure 1:**
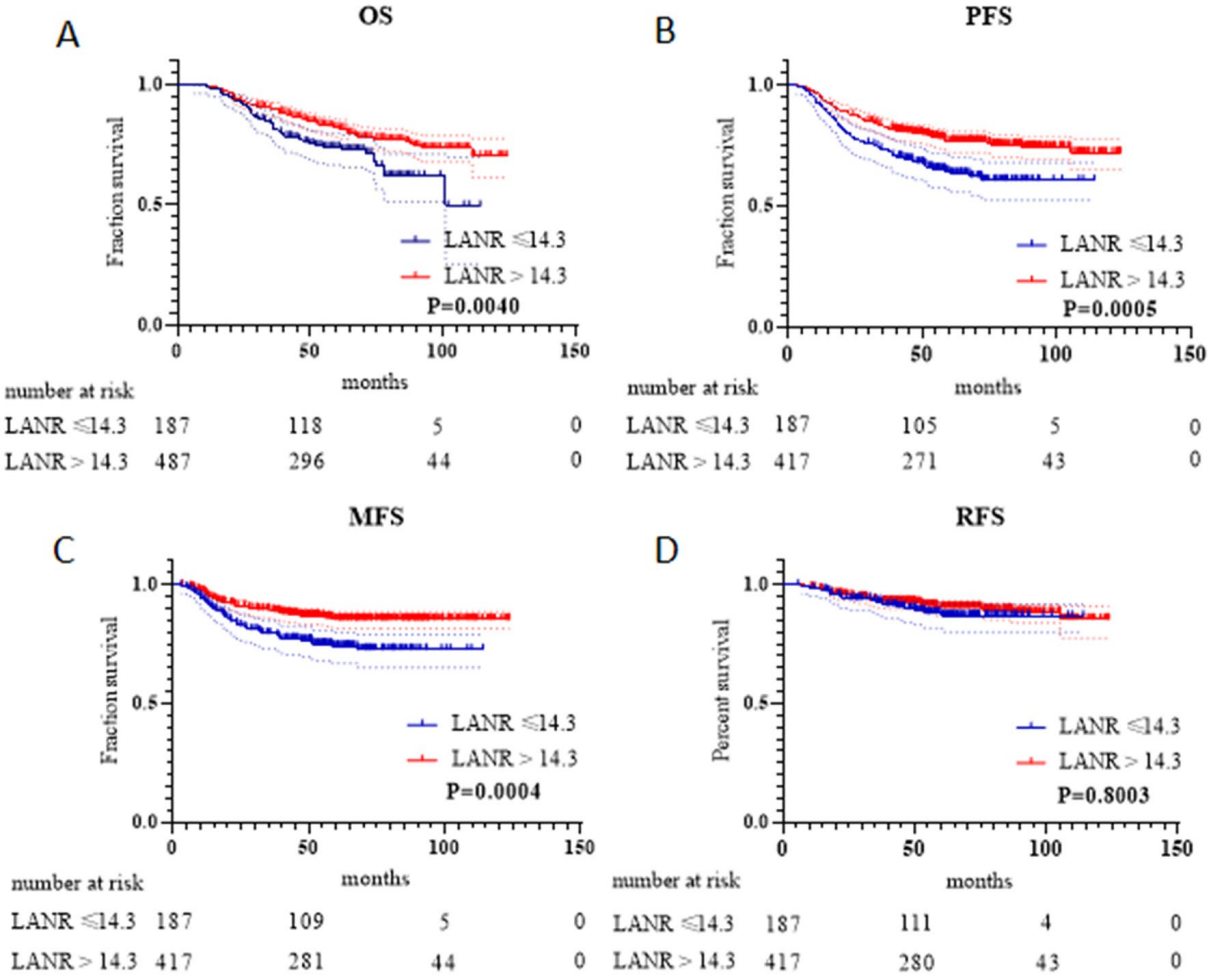
K‒M survival analyses of the LANR for (**A**) OS, (**B**) PFS, (**C**) MFS, and (**D**) RFS. *OS* overall survival, *PFS* progression-free survival, *MFS* metastasis-free survival, *RFS* relapse-free survival.

**Figure 2:**
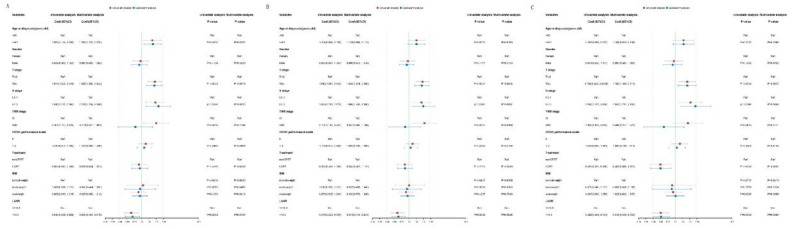
Univariate and multivariate Cox analyses of clinicopathological characteristics on (**A**) OS, (**B**) PFS, and (**C**) MFS in the training cohort of NPC patients. *OS* overall survival, *PFS* progression-free survival, *MFS* metastasis-free survival, *RFS* relapse-free survival.

K‒M analysis and the log-rank test showed that the LANR was significantly associated with OS, PFS, and MFS (Fig. 1). In addition to the LANR, age, T stage, N stage, and clinical stage were associated with patient OS in the training cohort (Fig. 2A). T stage, N stage, and clinical stage were associated with patient PFS (Fig. 2B). T stage, N stage, and treatment modality were associated with patient MFS. (Fig. 2C).

**Figure 3:**
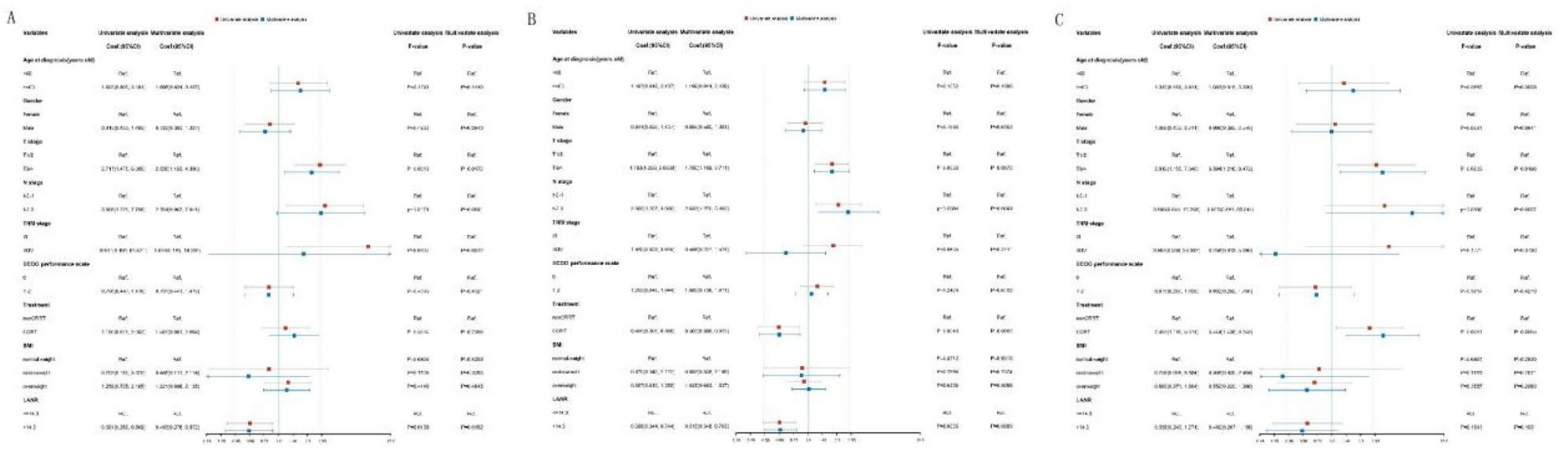
Univariate and multivariate Cox analyses of clinicopathological characteristics on (**A**) OS, (**B**) PFS, and (**C**) MFS in the validation cohort of NPC patients. *OS* overall survival, *PFS* progression-free survival, *MFS* metastasis-free survival, *RFS* relapse-free survival.

The variables that were significantly correlated with patient prognosis according to univariate analysis were incorporated into multivariate analysis. Cox regression analysis revealed that LANR (HR 0.650, 95% CI 0.460–0.919, P = 0.015), age (HR 1.700, 95% CI 1.122–2.575, P = 0.012), T stage (HR 1.828, 95% CI 1.265–2.642, P = 0.001), and N stage (HR 2.228, 95% CI 1.239–4.009, P = 0.008) were independent prognostic factors for OS (Fig. 2A). The LANR (HR 0.613, 95% CI 0.446–0.843, P = 0.003), T stage (HR 1.846, 95% CI 1.314–2.595, P < 0.001), and N stage (HR 1.999, 95% CI 1.183–3.380, P = 0.010) were independent prognostic factors of PFS (Fig. 2B). LANR (HR 0.515, 95% CI 0.348–0.763, P < B0.001), T stage (HR 1.780, 95% CI 1.169–2.711, P = 0.007), N stage (HR 2.633, 95% CI 1.270–5.462, P = 0.009), and treatment modality (HR 0.503, 95% CI 0.308–0.823, P = 0.006) were independent prognostic factors for MFS (Fig. 2C). The results found in the training cohort were further proven in the validation cohort (Fig. 3).

### Nomogram construction and validation

**Figure 4:**
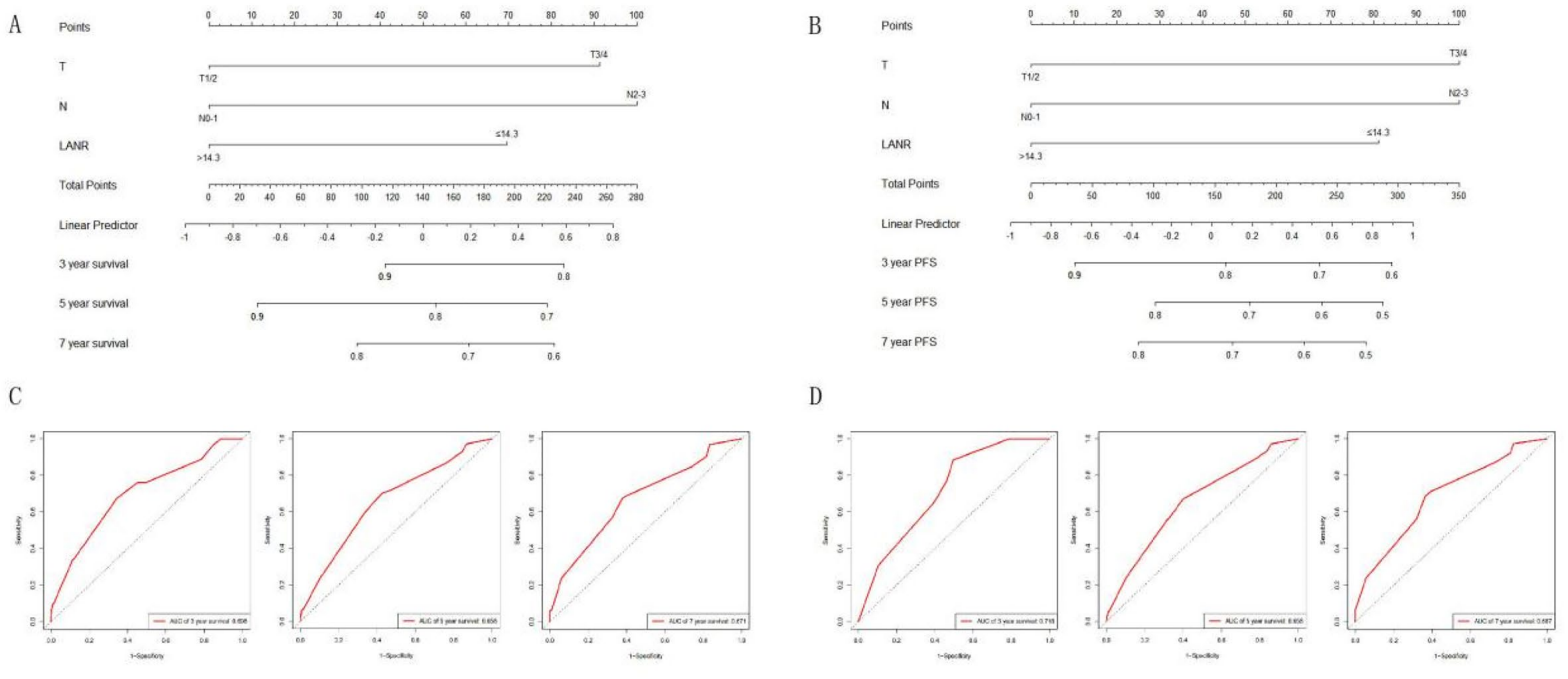
Nomograms for predicting 3-, 5-, and 7-year survival probabilities in the training cohort of NPC patients. (**A**) Nomogram incorporating the LANR and other clinicopathological characteristics for OS prediction in the training cohort; (**B**) nomogram incorporating the LANR and other clinicopathological characteristics for PFS prediction in the training cohort; (**C**) ROC curves for the nomogram for predicting OS in the training cohort; (**D**) ROC curves for the nomogram for predicting PFS in the training cohort. *OS* overall survival, *PFS* progression-free survival.

**Figure 5:**
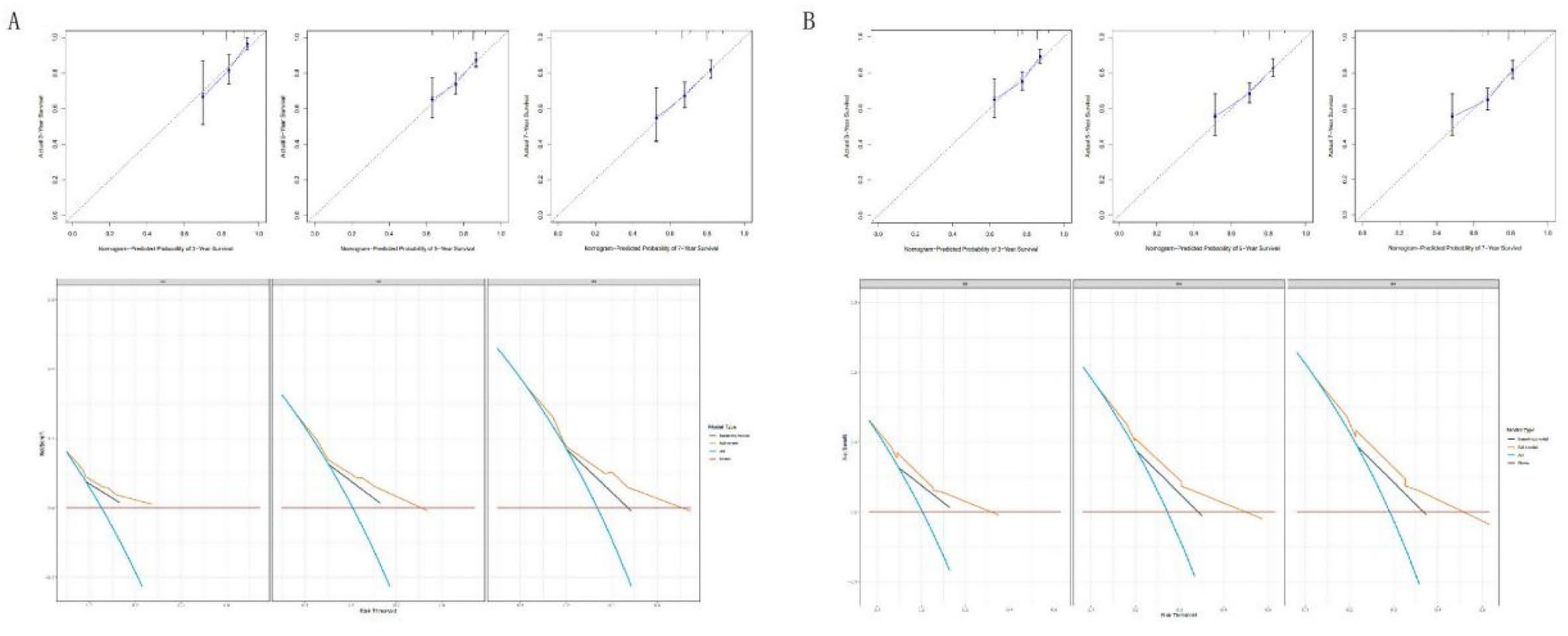
Calibration curves and DCA curves. (**A**) Calibration and DCA curves of the nomogram for predicting OS in the training cohort; (**B**) calibration and DCA curves of the nomogram for predicting PFS in the training cohort. *DCA* decision curve analysis.

**Figure 6:**
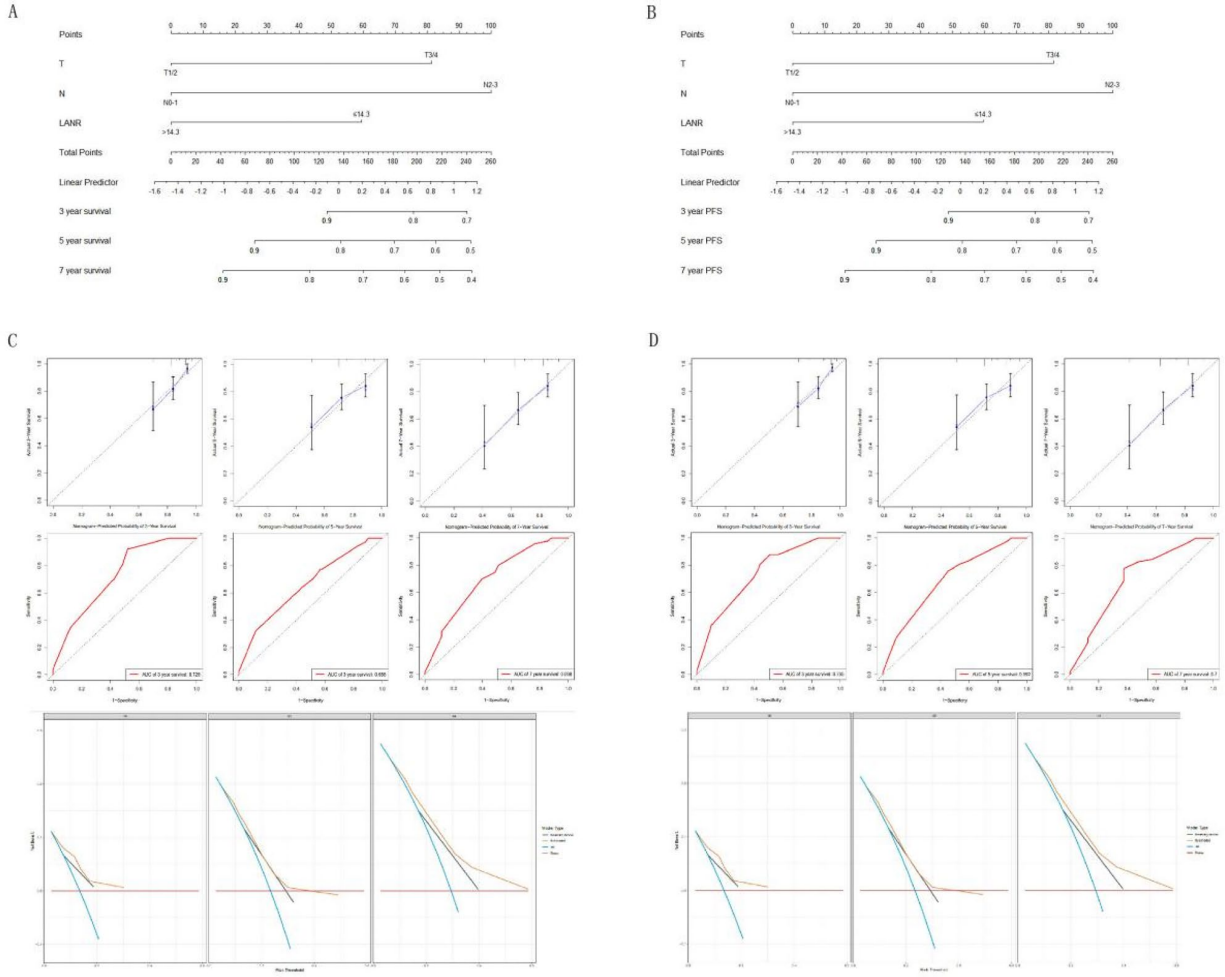
Nomograms, calibration curves and DCA curves in the validation cohort of NPC patients. (**A**) Nomogram incorporating the LANR and other clinicopathological characteristics for OS prediction in the validation cohort; (**B**) nomogram incorporating the LANR and other clinicopathological characteristics for PFS prediction in the validation cohort; (**C**) ROC curves, calibration curves and DCA curves for the nomogram for predicting OS in the validation cohort; (**D**) ROC curves, calibration curves and DCA curves for the nomogram for predicting PFS in the validation cohort. *DCA* decision curve analysis, *ROC* receiver operating characteristic.

The nomogram models of the training cohort were established based on the variables that were significantly correlated with OS and PFS (Fig. 4A,B). The area under the curve (AUC) values for 3-, 5- and 7-year OS were 0.698, 0.658, and 0.671, respectively (Fig. 4C). The area under the curve (AUC) values for 3-, 5- and 7-year PFS were 0.718, 0.658, and 0.687, respectively (Fig. 4D). The C-indices of the nomogram models for OS and PFS were 0.729 and 0.72, respectively. The calibration plots and DCA curves for the probability of OS (Fig. 5A) and PFS (Fig. 5B) showed excellent consistency between the nomogram model predictions and actual observations. The nomogram models were further validated in the validation cohort. The models also exhibited excellent predictive ability, with C-indices of 0.694 and 0.695 (Fig. 6).

## Discussion

In the present study, we retrospectively analyzed the prognostic value of a novel immune-nutritional index (LANR) and other clinical parameters in a cohort of 805 NPC patients and demonstrated that the LANR is an independent prognostic factor for OS, PFS and MFS. Furthermore, a nomogram combining the LANR and other independent prognostic factors (T stage, N stage, Eastern Cooperative Oncology Group [ECOG] score and treatment modality) was established, and the C-index and calibration curve showed robust predictive performance and good prognostic accuracy in both the training and validation cohorts.

The TNM staging system is the most widely used prognostic system for NPC patients. However, the mere use of TNM staging is not sufficient for stratifying patients due to the heterogeneity of cancer and the host. Therefore, identifying biomarkers to facilitate more accurate patient stratification is an unmet clinical need. Systemic inflammation is widely accepted as a hallmark of cancer^4^ and plays important roles in the development and progression of cancer cells as well as the prognosis of cancer patients. Lymphocytes are the main mediators of host antitumor immunity. The level of CD8+ T cells infiltrating the microenvironment is considered to represent host antitumor immunity and is correlated with increased sensitivity to immune therapy and improved prognosis^13,14^. Past reports have shown that lymphopenia induced by treatment, including radiation therapy, chemotherapy and surgery, is associated with a worse prognosis in patients with various cancers^15–17^. Neutrophils are another immune cell involved in antitumor immunity. Neutrophils can inactivate T cells by secreting various cytokines, resulting in tumor immune evasion and progression^18–20^. Moreover, tumor-infiltrated neutrophils can promote tumor angiogenesis^21^, and tumor-neutrophil crosstalk sustains prolonged tumor activation^22^. Hence, various blood-based immunity-related biomarkers, including the neutrophil–lymphocyte ratio (NLR), platelet–lymphocyte ratio (PLR), and systemic immune-inflammation index (SII), have been reported to be independent prognostic factors in cancer patients^23–25^. An elevated NLR is associated with worse survival in patients with various cancers, including NPC^26,27^. The underlying mechanism between the NLR and survival is partly mediated by CD3+ T-cell infiltration^28^.

Malnutrition is a common complication of tumors and is specifically prevalent in head and neck cancers^29^. The mechanism underlying cancer-related malnutrition includes reduced intake caused by cancer-related symptoms, treatment toxicity, excess catabolism and systemic inflammation. The serum ALB concentration is the most classical index of nutritional status. A decreased level of ALB is correlated with excess systemic inflammation that affects cancer progression^30^. The role of malnutrition as a prognostic factor for cancer patients has been widely explored. Several clinical studies have demonstrated that the serum ALB concentration is an independent prognostic factor in several malignant tumors^31–33^.

In the present study, we established a novel immune and nutritional index, the LANR, that is more comprehensive than a single immune or nutritional index. The prognostic importance of the LANR has been reported in several clinical studies. Liang et al.^11^ reported that a low preoperative LANR was a predictor of worse overall and progression-free survival in resectable colorectal cancer patients. Moreover, Takashi Oshima et al. reported that the LANR was an independent prognostic factor for gastric cancer^34^ and gastroesophageal junction cancer^35^. In our study, we also demonstrated that the LANR was an independent prognostic factor for OS and PFS in NPC patients. Moreover, we found that the influence of the LANR on NPC patient prognosis mainly relies on distant metastasis, not on local relapse, which indicates that immune and nutritional status mainly affect cancer metastasis and that the underlying mechanism is worth further exploration.

There are several limitations in our study. First, this was a single-center retrospective study, which will inevitably introduce bias to the results. Although we established a validation cohort for internal validation, external validation is lacking, which impairs the robustness and generalizability of the study. Multicenter research is planned for further validation of the results of the present study. Second, several other prognostic factors were not included in the analysis. For example, the EBV-DNA copy number is generally accepted as a prognostic factor in NPC patients. However, a standard examination procedure has not yet been established, which makes the results from different centers incomparable. Due to the different testing methods used at our center and the missing data for a proportion of patients, these patients were not included in our analysis. Therefore, we could not compare EBV-DNA and LANR, but such an investigation would be a worthwhile aspect in future research.

## Conclusions

In summary, a low pretreatment LANR is related to a worse prognosis and is an independent factor for OS, PFS and DMFS in NPC patients. Moreover, the NRS-2002 is an effective, convenient, and economical marker that provides a reference for prognostic stratification and new treatment strategies for NPC patients.

### Supplementary Information

Supplementary Figure S1.

## Data Availability

The raw data supporting the conclusions of this article will be made available by the corresponding author (email address: houtao@csu.edu.cn) without undue reservation.
