# Supplementary figures and images for: Nomograms based on the lymphocyte–albumin–neutrophil ratio (LANR) for predicting the prognosis of nasopharyngeal carcinoma patients after definitive radiotherapy
Source: Sci Rep. 2024 Mar 5;14:5388. doi: 10.1038/s41598-024-56043-z (PMC10915143; doi:10.1038/s41598-024-56043-z)

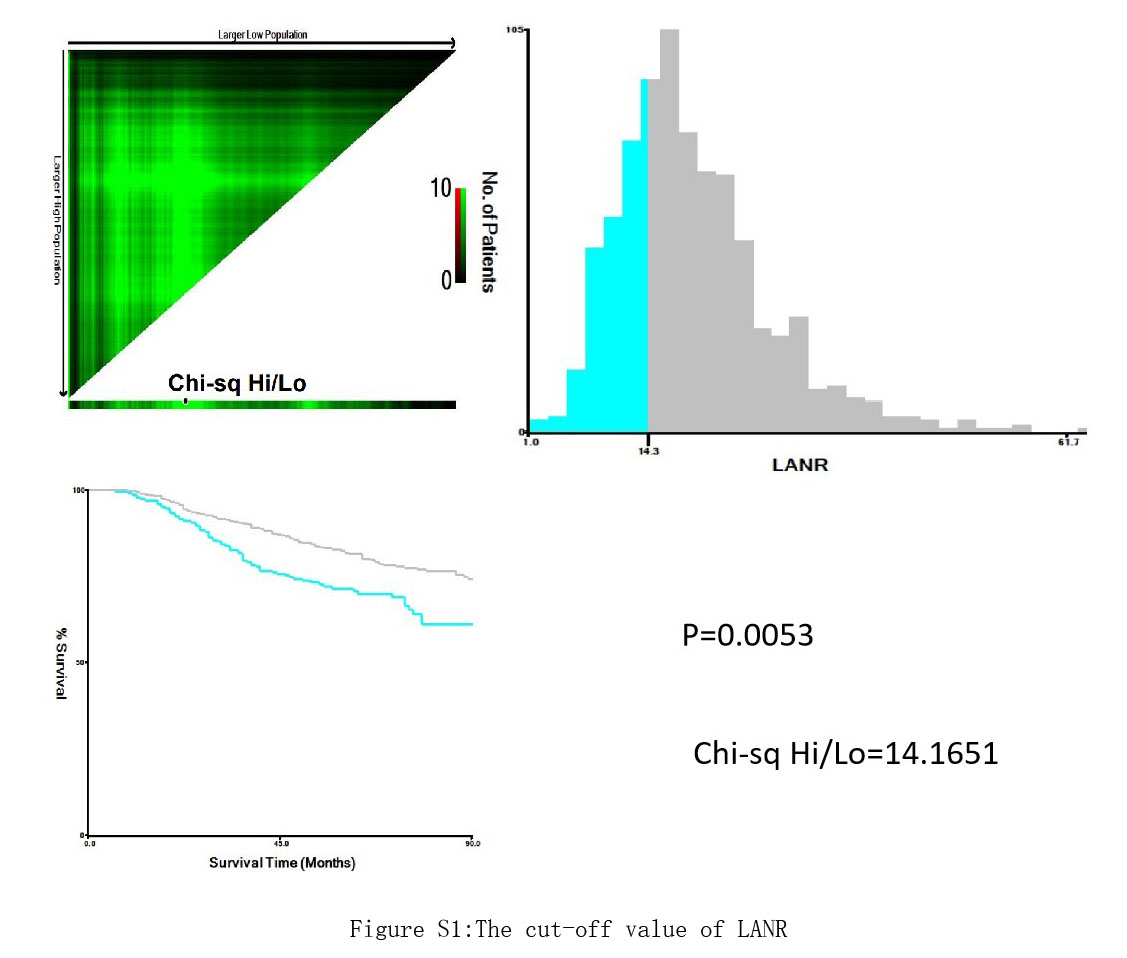

Supplement: Supplementary file 1 — Supplementary Figure S1. [file 41598_2024_56043_MOESM1_ESM.jpg]
